# Vitamin D intoxication presenting as acute renal failure

**DOI:** 10.4103/0971-4065.43693

**Published:** 2008-07

**Authors:** M. A. Naik, K. A. Banday, M. S. Najar, A. R. Reshi, M. A. Bhat

**Affiliations:** Department of Nephrology, Sher-I-Kashmir Institute of Medical Sceinces, Soura, Srinagar, India

**Keywords:** Vitamin D, acute renal failure, hypercalcemia

## Abstract

A 70 year-old male presented with acute renal failure and mental obtundation. On examination, he was found to have hypercalcemia and on further questioning, it was found that it was secondary to injections of a slow-release vitamin D preparation. Although total body exposure is sufficient for vitamin D synthesis, increased vitamin D deficiency secondary to poor exposure to sunlight is observed in some parts of the world. We report here a case of vitamin D intoxication from the Kashmir valley where vitamin D deficiency is 100% in the general population that is confined indoors.

## Introduction

Vitamin D toxicity is a known cause of hypercalcemia and renal failure. The daily requirement of vitamin D is about 200–600 IU and the skin can only produce around 10,000 IU of vitamin D after total body exposure to UV light.^1^ Although vitamin D has a wide therapeutic index, its toxicity is well known and cases of accidental ingestion, self medication, and malpractice have been reported. We report here a case of malpractice-related vitamin D intoxication in an elderly male who presented with hypercalcemia, acute renal failure, and mental obtundation.

## Case Report

Our patient was a 70 year-old male hypertensive who had been undergoing treatment (enalapril, 5 mg) for the last three years and was nondiabetic. He presented with lethargy, loss of appetite, constipation, confusion, and pain in the abdomen. Examination revealed a pulse of 76/min, blood pressure of 130/80 mm Hg, and no systemic abnormalities. Routine chemistry revealed; Hb 12 g/dL, TLC 6.1 × 10^9^/L, DLC: N 64 %, L 24%, platelet 123 ×10^9^/L, ESR 12/1^st^ h, urea 87 mg/dL, creatinine 3.8 mg/dL (0–1.5 mg/dL), glucose 98 mg/dL, serum calcium 13.5 mg/dL (9.5–11.5 mg/dL), serum phosphorus 8.6 mg/dL (3.5–5.5 mg/dL), uric acid 6.6 mg/dL, LDH 292 U/L, total protein 7.0 g/dL, albumin 4.0 g/dL, bil 0.75 mg/dL, OT 35 U/L, PT 40 U/L, ALP 210 U/L. Urine: normal, 24 h urinary proteins 0.15 g/day, the 24 h urinary calcium 200 mg/dL, chest X-ray: normal, electrocardiography normal, the ultrasound was normal.

The patient was managed by continuous saline infusion, diuretics, hydrocortisone, and bisphosphonates. The level of serum calcium on the 10^th^ day of treatment was 10.28 mg/dL, that of phosphorus was 3.94 mg/dL, and the creatinine level stabilized at 1.5 mg/dL.

The patient reported that he had been taking injection vitamin D, 6,00,000 IU (OSTA D3) every two weeks for the last two years for arthralgias and generalized body aches. He had received 35 injections of vitamin D to date, with a cumulative dose of 2,10,00,000 IU.

The PTH level was 15 ng/L (10–60 ng/L) and the 25-hydroxy vitamin D level was 302 nM (47–144 nM). The PSA was normal and the myeloma profile was negative. The serum calcium and renal functions remain normal on follow up.

## Discussion

Vitamin D intoxication can be accidental^2^ or due to self medication,^3^ after the topical application of vitamin D ointment,^4^ induced by dietary or OTC supplements,^5^,^6^ or iatrogenic in some unusual cases.^7^ The clinical manifestations of this intoxication are kidney disorders (65.0%), renal insufficiency (51.0%), gastrointestinal tract disorders (23.0%), and arterial hypertension (52.0%).^8^

The overzealous use of vitamin D in European countries due to the fear of rickets can lead to vitamin D intoxication;^3^ although some cases have also been reported from India.^9^ The season, the geographic latitude, the time of day, cloud cover, smog, and sunscreen affect UV exposure and vitamin D synthesis.^10^ For example, sunlight exposure from November through February in Boston is insufficient to produce significant vitamin D synthesis in the skin. However, in a country like India, sunlight exposure is sufficient for vitamin D synthesis except for the northern part of India.

In the Kashmir valley, the prevalence of vitamin D deficiency is quite high, 69.6% in individuals exposed to the outdoors, to 100% in those confined indoors, reflecting the lower mean weekly exposure to sunlight.^11^

Our patient had received a slow-release preparation of vitamin D after every 15 days for the last two years, leading to a cumulative dose of 2,10,00,000 IU. This emphasizes the need to regularly assess the levels of vitamin D in patients suspected of its deficiency and who are put on vitamin D replacement therapy.
